# Carbon Fiber Reinforced Recycled Polypropylene/Polyolefin Elastomer Composites with High Mechanical Properties

**DOI:** 10.3390/polym16070972

**Published:** 2024-04-03

**Authors:** Jin Wei, Abdukeyum Abdurexit, Ruxangul Jamal, Tursun Abdiryim, Jiangan You, Zhiwei Li, Jin Shang, Qian Cheng

**Affiliations:** 1 State Key Laboratory of Chemistry and Utilization of Carbon Based Energy Resources, College of Chemistry, Xinjiang University, Urumqi 830017, China; 17793738494@163.com (J.W.); jayou@xju.edu.cn (J.Y.); shangjinpop@163.com (J.S.); qq1970755293@163.com (Q.C.); 2 State Key Laboratory of Chemistry and Utilization of Carbon Based Energy Resources, State Key Laboratory of Oil and Gas Fine Chemicals, Ministry of Education & Xinjiang Uygur Autonomous Region, College of Chemical Engineering Technology, Xinjiang University, Urumqi 830017, China; abdukaiyum@sohu.com (A.A.); jruxangul@xju.edu.cn (R.J.); li2812355161@163.com (Z.L.)

**Keywords:** carbon fibers, recycling, mechanical properties, thermal properties

## Abstract

The treatment of waste plastics has gradually become a hot topic in the current scientific community. In response to the needs for high-impact performance R-PP-based composites, carbon fiber (CF)-reinforced polyolefin elastomer (POE)/recycled polypropylene (R-PP) composite (CF/POE/R-PP) was prepared by the mechanical blending method, and its mechanical and thermal properties were systematically studied. It was found that the CF could effectively improve the bending and notch impact strength as well as enhance the thermal stability of POE/R-PP. Furthermore, a stable and dispersed composite interface formed by the combination of maleic anhydride-grafted polypropylene (PP-g-MAH) with the surface of CF and the fusion alkyl chains in R-PP and POE further enhanced the CF’s reinforcing effect. As a result, the addition of 9 wt.% CF successfully improved the heat resistance of the composite material, and the residual carbon content increased by 97.84% after sintering. The composite toughening of POE and CF effectively improved the impact strength of the composite material, with a maximum increase of over 1000%. This study ultimately resulted in a high-impact-resistant composite material.

## 1. Introduction

Polypropylene (PP) has high mechanical strength and good processing properties and is widely used in food and drug packaging [1,2,3]. Long-term use of PP generates a large amount of household waste, resulting in additional resource waste and environmental pollution [4,5]. Compared to PP, recycled polypropylene (R-PP) has poor mechanical and thermal properties due to possible molecular chain degradation during mechanical processing. Considering the environmental and performance issues, efficient modification of R-PP is valuable [6,7].

Common R-PP modification methods mainly relied on physical methods [8,9,10]. Jones et al. reported that the R-PPs have lower crystallinity and that the mechanical and thermal properties deteriorate compared to the original PP, but their elongation at the break increases, which is due to the plasticity effect of low-molecular-weight segments [11]. Kang et al. [12] prepared recycled composite materials with different cycles by grinding the initial short glass fiber-toughened composite material and the initial long glass fiber-reinforced composite material. AlMaadeed et al. mainly improved the mechanical strength of R-PP by mixing wood powder/glass fiber; this especially improved the tensile strength and Young’s modulus of R-PP [13]. Črešnar et al. [14] discussed the surface properties of composites filled with R-PP and wood fiber (WF) and proposed that the distribution and orientation of wood fiber filler in the R-PP matrix were the key factors affecting the overall surface properties of materials. Sascha Stanic et al. [15] mainly studied composites made of two long-chain branched polypropylenes (LCB-PP) and five linear polypropylenes (L-PP) and the relevant dynamic rheological mechanisms.

Polyolefin elastomer (POE) is a common reinforcing phase used to enhance the toughness of resins. POE provides mechanical support and flowability for composite materials due to its molecular structure, which contains both long-compliant and short-branched chains. Yang et al. [16] studied the brittle–ductile transition of PP/POE composite materials and pointed out that, due to the flow index of PP itself, a significant increase in toughness can only occur when the mass fraction of POE reached the threshold. Wang et al. [17] prepared a novel magnetic super toughened PP composite material using maleic anhydride grafted ethylene octene copolymer (POE-g-MAH) and reactive nano Fe_3_O_4_ through one-step melt blending method, achieved a notch impact strength of 74.2 kJ/m^2^. Yue et al. [18] successfully prepared an environmentally friendly high water absorption composite material using recycled waste plastics including R-PP, POE, and super-absorbent polymer (SAP) composites.

Carbon fiber (CF) is a high-strength, high-modulus material commonly used for modification. Andoko et al. [19] used CF and Ceiba petandra fiber (CPF) to reinforce polymer composites to create lightweight, high-performance composites, and it was found that, with the 15 wt.% CF and 15 wt.% CPF, the hybrid composite showed the best mechanical properties, with the tensile and flexural strength reaching 130.16 ± 17.37 MPa and 98.53 ± 12.2 MPa, respectively. Kawasaki et al. [20] mainly focused on the influence of temperature distribution during the CF/PP ultrasonic welding process, and the study confirmed the practical application of CF in composite materials. Kwon et al. [21] also studied the practical application of CF in PP composite materials.

In the present work, the carbon fiber-reinforced polyolefin elastomer/recycled polypropylene composite material (CF/POE/R-PP) was prepared using mechanical melt blending, in which the R-PP was the matrix, with POE and CF as the reinforcement phases and polypropylene-graft-maleic anhydride (PP-g-MAH) as the compatibilizer, respectively. The main focus of this study was investigating the effect of CF/POE on the impact resistance of the R-PP-based composite material and identifying the potential mechanism of synergistic toughening through chemical structure analysis. The combined toughening of POE and CF greatly increased the impact strength of the composites, the addition of 9 wt.% CF successfully improved the heat resistance of the composites, and the carbon residue content increased by 97.84% after sintering. This study provides valuable insights for the modification of R-PP to create potential value in practical applications.

## 2. Materials and Methods

### 2.1. Materials

R-PP (0.90 g/cm^3^, melt index 15 g/10 min, Rockwell hardness 98, softening point 125 °C) and PP-grafted maleic anhydride (PP-g-MAH, Graft rate 1.0%, Melt index 80–100 g/10 min) [22,23] were procured from Dongguan Hongxing New Materials Company (Dongguan, China), while POE was obtained from Suzhou Yinglun Er Engineering Plastics Company (Suzhou, China). To enhance the overall strength of the material, micron-sized CF (Density: 1.75 g/cm^3^, diameter: 7 µm) from Carbon Technology (Shenzhen, China) company was utilized. The specific formula proportions are detailed in Table 1.

### 2.2. Sample Preparation

**Figure 1:**
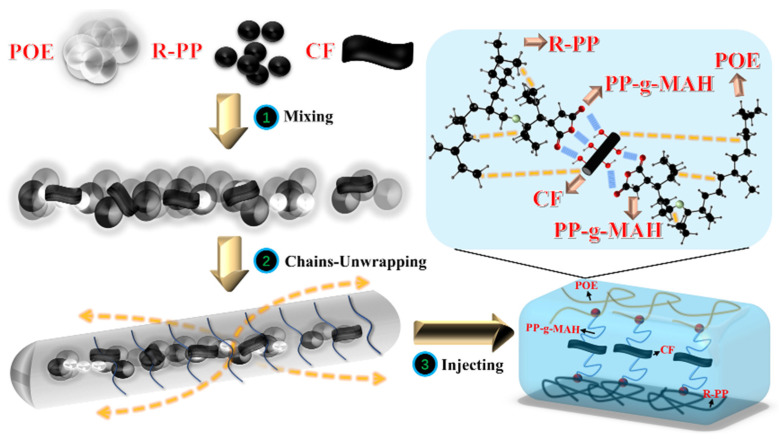
The processing process of composite materials.

A co-rotating twin-screw extruder (model TE-20) with a length-to-diameter ratio (L/D) of 32, screw diameter of 21.7 mm, and main motor power of 7.5 kW was utilized. Following the detailed formula, R-PP and other raw materials such as POE and CF were thoroughly mixed through the twin-screw extruder, with temperature control set at each section ranging from 175 °C to 180 °C (180 °C–185 °C–185 °C–180 °C–180 °C), host frequency at 9.0 Hz, and feeding frequency at 8.0 Hz. Crushers were employed for crushing and granulation, while vacuum dryers were used for drying at approximately 80 °C for about 8 h. Subsequently, twin-screw injection molding machines were used for molding, followed by a 36-h room temperature treatment to eliminate internal stress. The possible processing process is depicted in Figure 1.

### 2.3. Methods

#### 2.3.1. Density

Measurements were conducted using an electronic density meter, where calibration was initiated by resetting to zero and entering calibration mode. Samples were clamped onto the measurement table using tweezers for density testing in air. Following this step, samples were cleaned with alcohol and placed in de-ionized water to determine their density in water. Subsequently, the machine automatically calculated the density-specific gravity value based on the density ratio in the two environments and cycled through testing a minimum of five samples.

#### 2.3.2. The Mechanical Testing

After relieving internal stresses, tensile tests, flexural tests, and notched impact tests (notches machined to a depth of 2 mm by a notch sampling machine) were conducted on the composite materials, with the average values taken from five samples to ensure data reliability. The sample dimensions used in the tensile test were 80 mm × 13 mm × 2 mm. For the flexural test, sample dimensions of 65 mm × 13 mm × 5 mm were used, and for the impact test, sample dimensions of 80 mm × 10 mm × 4 mm were employed. The tests were performed using the ISO 527 [24] testing method, with a dumbbell-shaped specimen and a gauge length of 20 mm. The initial distance between the fixtures was set at 50 mm, and the tensile properties were measured at a crosshead speed of 50 mm/min at room temperature.

#### 2.3.3. Scanning Electron Microscopy (SEM)

The microstructure of composite materials was studied using the Germany Zeiss Sigma 300 scanning electron microscope (instrument grade, Carl Zeiss AG, Oberkochen, Germany). Due to the poor conductivity of the R-PP matrix, a sputter-coating method was employed to obtain clear images, focusing on the microstructure and dispersion state of the multiphase. The fractured surface after impact testing was polished, cross-sectioned, placed on a holder, securely attached with conductive adhesive tape, and sputter-coated with gold powder evenly. The testing mode was secondary electrons, with magnifications of 10,000×, 5000×, 2500×, 1000×, and 500×.

#### 2.3.4. Thermal Differential Scanning Calorimetry (DSC)

The melting and crystallization behavior of R-PP/POE/CF composite materials were investigated using the TA DSC250 (New Castle, DE, USA) under a protective gas (nitrogen) atmosphere. The temperature was ramped from room temperature to 350 °C, then lowered to room temperature and raised again, using the same heating and cooling rate (10 °C/min). The degree of crystallinity was calculated using the following formula:

(1) $$X_{c}\left(\%\right)=(∆H_{m}/\varphi_{R-\mathrm{PP}}\cdot ∆H_{m}^{0})\cdot 100\%$$

where $∆H_{m}$ represents the melting enthalpy of the sample (J/g), $∆H_{m}^{0}$ = 207 J/g represents the melting enthalpy of 100% crystalline PP [25], and $\varphi_{R-\mathrm{PP}}$ represents the R-PP content in the samples.

#### 2.3.5. Thermogravimetry (TGA)

To test the thermal stability of the composite materials, a thermal gravimetric analysis (TGA) was conducted using the Germany Netzsch TG 209 F3 Tarsus (NETZSCH, Selb, Germany) under a nitrogen atmosphere. Approximately 6 mg of the sample was placed in a ceramic crucible and heated from room temperature to 800 °C at a rate of 10 °C/min.

#### 2.3.6. Fourier Transform Infrared (FT-IR) Spectroscopy

FT-IR analysis was performed using the VERTEX 70 RAMI (Bruker Corporation, Karlsruhe, Germany). The spectral resolution was set at 16 cm^−1^, with a wavelength range of 4000 cm^−1^ to 400 cm^−1^. The plastic products were cut into small particles and pressed with potassium bromide with a weight of approximately 5 mg.

#### 2.3.7. X-ray Diffraction (XRD) Testing

Testing was conducted using the Germany Bruker D8 (Bruker Corporation, Karlsruhe, Germany) Advance with a copper target. The scanning range was 5–90° with a scan rate of 2°/min.

## 3. Results and Discussion

### 3.1. Chemical Structure Analysis

**Figure 2:**
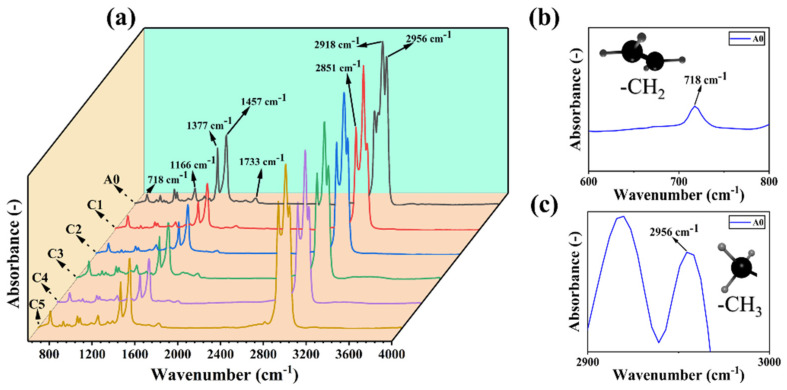
(**a**) The FT-IR spectra of A0 and C system, (**b**) FT-IR spectrum of the vibration peak for -CH_2_ of A0, (**c**) FT-IR spectrum of the vibration peak for -CH_3_ of A0.

Fourier transform infrared spectroscopy (FT-IR) was used to analyze the chemical structure and interactions of the composite materials, ensuring the accuracy of their compositions. As shown in Figure 2, the R-PP and the R-PP/POE/CF have similar peaks, which mainly result from the polymer backbone of PP and POE [26,27,28,29]. The main specific peaks for R-PP are shown at 2956 and 1377 cm^−1^, while the main specific peaks for POE are shown at 2851 and 718 cm^−1^. The peaks at 2956 cm^−1^ and 2851 cm^−1^ are the symmetrical and asymmetrical stretching vibrations of the C–H bonds, such as CH_2_ and CH_3_. The peak at 1377 cm^−1^ is assigned to the CH_2_ and CH_3_ transformation oscillations of aliphatic groups in the polymer chain. The peak at 718 cm^−1^ is associated with the CH_2_ rocking vibrations of macromolecules. To compare with that of R-PP, the relative peak intensities at 2851 cm^−1^ and 718 cm^−1^ in R-PP/POE/CF are higher, indicating the presence of POE.

**Figure 3:**
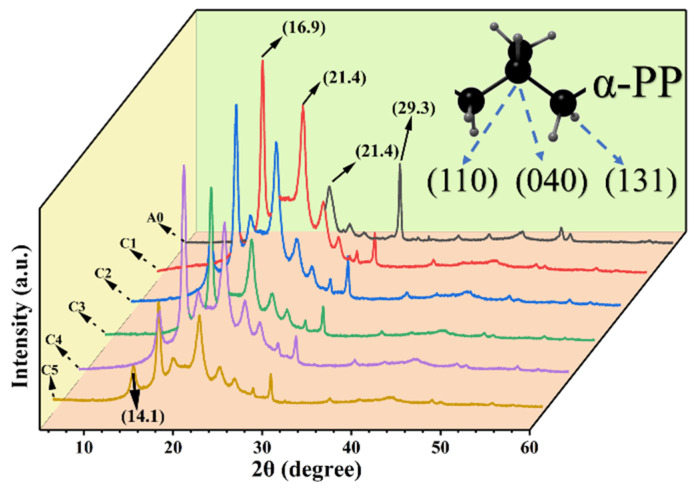
XRD curves of A0 and C system.

Figure 3 shows the results of XRD, which is commonly used to observe crystal peaks or determine the chemical structure in polymers. The results from FT-IR have speculated that the matrix in composite is R-PP, and the crystal structure of the R-PP and the R-PP/POE/CF need to be explored by XRD. The curves show that both the A0 (R-PP) and C series (composite) samples exhibit peaks around 14.1°, 16.9°, and 21.4°, which are assigned to the (110), (040), and (131) crystal planes, respectively. We continued to speculate that the A0 (R-PP) and C series (composite) may belong to α-PP [30,31], and XRD testing shows that there is no β-PP crystal configuration in the A0 and C system [32], from which it can be understood that the α-PP is the most stable and classic PP crystal form. From this, combined with FT-IR analysis, it can be concluded that the A0 and C series are indeed PP substrate materials, and the appearance of impurity peaks represented by 29.3° (sharp peaks) confirms that the R-PP material is similar to what is expected and is a self-blend. Due to direct production without any sorting and process limitations, such as stretching and extrusion during the processing, the recycled materials produced have extremely poor crystalline phases, which can usually be attributed to impurity nucleation.

### 3.2. Thermal Profile

**Figure 4:**
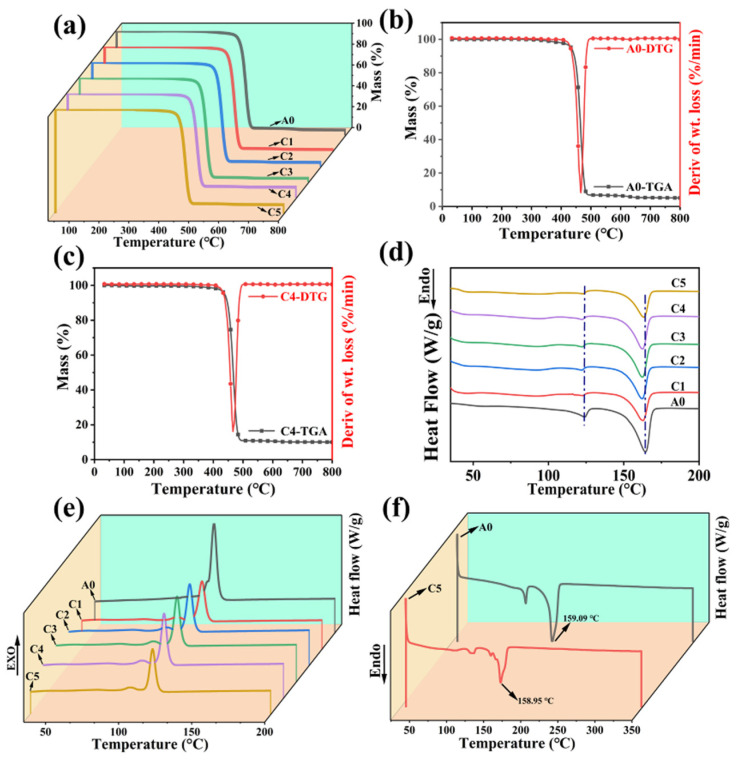
The curve of A0 and C series TGA (**a**), the DTG integration curve of A0 and C4 (**b**,**c**), the first heated and first cooling curve of A0 and C series (**d**,**e**), and A0 and C4 for the second heated curve (**f**).

Figure 4 shows the thermodynamic analysis of the composite material, in which the single step degradation process experienced by different components of the composite material can be observed. Figure 4a shows the TGA test, indicating that the actual initial decomposition temperature (tangent extrapolation point) increases when CF is added to the composite material. The decomposition temperature of C4 increases from 450 °C to about 458.2 °C, which indicates that the composites have higher thermal stability compared to R-PP. After adding CF, the ablation resistance gradually improves. The residual carbon mass fraction of A0 is 5.09 wt.%, while the residual carbon weight fraction of C4 at 800 °C is 10.07 wt.%. Overall, the addition of CF makes an important contribution to improving the thermal stability of the blend. The TGA experiment fully demonstrates the positive significance of micron-level CF, even with a low additive content. The important labeling data of TGA and DTG related to the A0 (R-PP) and C series (Composite) are detailed in Table S2. Figure 4d,e show the first temperature rise and fall curves of the C system, respectively. They represent the basic Tm peak of R-PP, approximately 165 °C [33]. After calculation, it is found that with the addition of CF, the crystallinity of the composite material improves to some extent and shows a regular upward trend. An increase in crystallinity usually leads to an increase in rigidity, but it also leads to a decrease in toughness, as crystallinity regulates the inter-chain arrangement of composite materials, causing them to move along constrained molecular chains and increasing their intermolecular forces. The melted peak at 125 °C can be understood as a PE impurity peak, and similar findings have been found in the work [34]. The DSC data related to the A0 and C series are detailed in Table S3. Figure 4f shows the melting point and crystallization temperature of the composite material after high-temperature annealing and elimination of the thermal–mechanical history. Table S4 reflects the secondary heating curves and related physical parameters of A0 and C5 after return. Compared with the original room temperature crystallinity, there is no significant change in crystallinity. The crystal structure of R-PP is incomplete and usually contains crystal defects. The growth process of new crystals essentially relies on the adhesion of carbon chains by molten materials to the continuously formed surface of the original crystals. This is due to the influence of atomic thermal motion or other conditions during the actual crystal growth process, and the arrangement of atoms may cause the region to deviate from the ideal crystal structure. Even after adding micron-level CF to the low filler, the overall melting point still maintains a downward trend, which is due to the blocky defects in the matrix lattice caused by it. After damaging the crystal cell and further leading to pores or cracks, microscopic substances further affect the thermal conductivity between phases, leading to uneven heating of various components, mainly uneven heat transfer and diffusion ability. The local state changes after passing through multiphase points and becomes the melting core, which is the beginning of the melted process. However, the presence of multiphase components in composite materials widens the melting limit region, and this validates the DSC curves changes of Figure 4d,f.

### 3.3. Mechanical and Surface Profile

**Figure 5:**
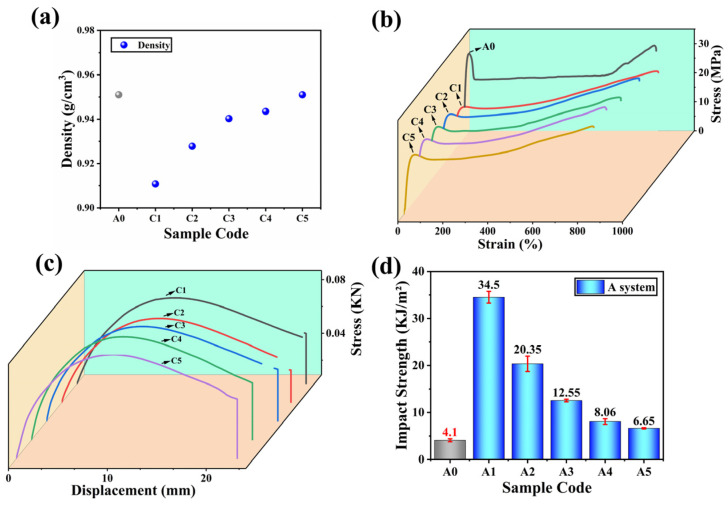
(**a**) Density results of the A0 and C system, (**b**) Stress–Strain curves of the A0 and C system, (**c**) Stress–Displacement about bending properties of the A0 and C system, (**d**) the Impact Strength of the A0 and C system.

Figure 5a shows the results of the sample density. The main purpose of this experiment was to measure the basic physical properties of the mixture and provide reliable parameters for further mechanical and physical performance analysis. It can be seen that the density of the composite material is positively correlated with the volume fraction of CF added. This may be due to the addition of compatibilizers, which mainly improves the uniformity of the blend by improving the adhesion between phases, thereby further increasing the density of the composite material. This indicates that the R-PP/POE/CF blend in this experiment belongs to lightweight or relatively lightweight composite material. The density of a material determines the upper limit of its specific usage range. Figure 5b shows that with the increase of POE content, the overall strength and modulus of the composite material decrease compared to R-PP, while the elongation at the break shows an increasing trend to varying degrees. The material has a large plastic deformation space before fracture. This usually means that it has better scalability. It should be pointed out that intentionally designed or adjusted POE weight percentages have a significant impact on the mechanical properties of composite material systems. Figure 5c shows the bending performance of the C series composite material, with specific data shown in Table S2. The main disadvantage of POE is that it significantly reduces the bending strength of the composite material. Compared to A0, the bending strength of C1 decreases by about 65.89%. The addition of CF effectively improves the bending strength and continues to increase with the increase of the CF filling rate. The bending strength of C5 is 53.92% higher than that of C1. CF has excellent effectiveness in strength and modulus. Figure 5d shows the impact strength of the composite materials, respectively. In system C, the impact strength increases twofold with the increase of CF. Compared with A0, it increases from 4.10 MPa to 33.39 MPa and finally increases to 46.96 MPa (AVG), with a maximum increase of over 1000%. The impact strength of the composite materials would be further improved by CF. This is similar to stretching, where adding CF to different POE contents has a completely different overall effect on the composite material.

Preliminary speculation suggests that this is due to reaching the lower limit of the support threshold when the POE value increased to a certain proportion. At this point, CF begins to synergistically enhance the impact resistance of the R-PP-based composites. Due to the deformation of thermoplastic elastomers after stress concentration consumes a large amount of energy generated by external impact, it is difficult for microcracks to further diffuse after occurrence. Ultimately, it leads to a doubling of the impact strength. Related research provides an explanation for the intrinsic toughening mechanism of thermoplastic elastomers and their impact on the improvement of PP properties [35]. Mechanical performance analysis shows when CF is not added, R-PP/POE/MAH composite materials exhibit different impact resistance based on the proportion of POE in them, but they are all several times higher than R-PP. At the same time, the composite material is endowed with toughness characteristics, that is, the fracture elongation is further improved. The toughening effect of POE on R-PP mainly includes two aspects: first, as the center of stress concentration, POE guides the R-PP to produce a large number of crazes and shear zones. On the other hand, POE controls the development of crazes to terminate them in a timely manner, preventing them from developing into destructive cracks. The macroscopic manifestation of crazes is the phenomenon of stress whitening. However, the key to brittle ductile transition lies not only in the content of POE, but also in its fineness and dispersion. The enrichment or poor dispersion of POE is not conducive to the toughening of composite materials, Although PP-g-MAH was used in this study, its improvement in compatibility with various phases may mainly focus on PP/CF or POE/CF, which would be the fundamental reason for the ultimate decrease in impact strength of A and B systems. Therefore, it is crucial to ensure that all components are uniformly dispersed in the matrix when constructing composite materials.

**Figure 6:**
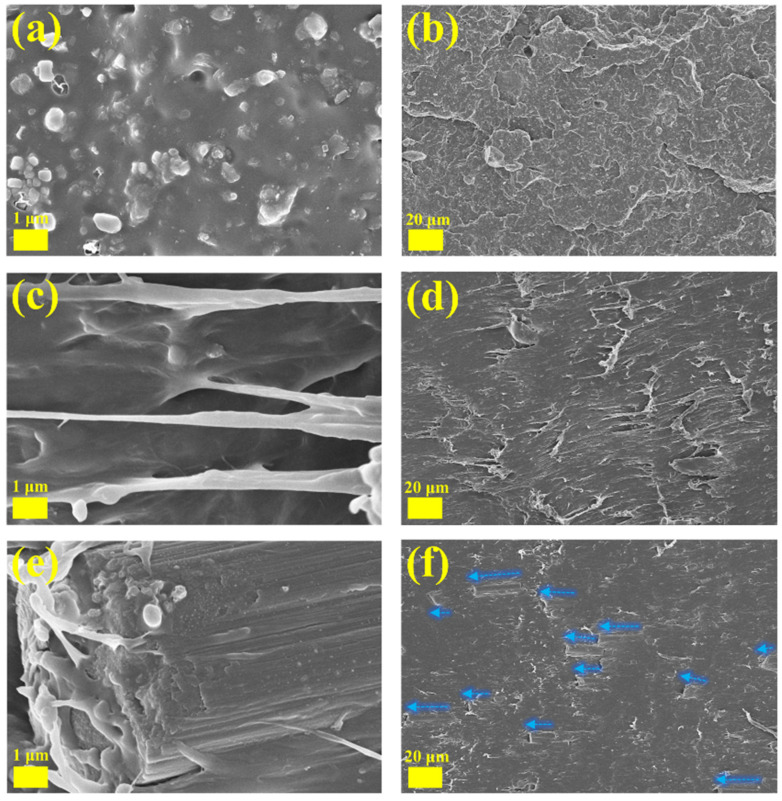
(**a**,**b**) The SEM of A0 at 500× and 10,000×, (**c**,**d**) the SEM of C1 at 500× and 10,000×, (**e**,**f**) the SEM of C5 at 500× and 10,000×. The blue arrow shows the possible layout direction of CF.

Figure 6 shows SEM images of the fracture surfaces of A0, C1, and C5 at different magnifications. Figure 6a,b indicate that R-PP itself is a mixture, and the impurities may be closely related to their sources in different types of plastic products. The appearance of pores may be due to weakly bound recycled components being pulled out of the matrix. After adding PP-g-MAH, the gaps and crack lines between multiphase spaces further decrease, and the adhesion between interfaces significantly increases. As shown in Figure 6d, C1 exhibits a significant tearing sensation after being subjected to external forces. This may be due to the hard segment of POE (physical crosslinking point) limiting further separation of the R-PP substrate, resulting in no significant voids and the formation of tear-like morphology.

Figure 6e,f show the microstructure of C5 at 500× and 10,000× magnification. The CF loaded on the fracture surface can be observed. It can be observed that under low load, the CF dispersed in the composite matrix appears to be arranged in the same direction, which would be directly related to the further improvement of the C system impact strength. The above phenomena observed in the electron microscope may be related to the mechanical performance results discussed earlier. A clear binding structure is formed between CF, POE, and R-PP, and the good compatibility between thermoplastic rubber and plastic is also reflected in the composite system. In addition, the addition of CF does not change the characteristics of local plastic deformation after impact; that is, there is no large-scale destructive deformation, which may also be an effective manifestation of preventing crack propagation and dissipating energy. Through the joint analysis of the SEM and mechanical strength results, it is speculated that POE mainly has an intrinsic toughening effect. After being impacted, it prevents the appearance of crack tips through deformation or silver lines. Fiber materials represented by CF will further consume impact energy through interfacial detachment after the appearance of the tips, thereby reducing the crack attenuation, further speeding up the blend, and ultimately improving the impact resistance energy. Simultaneous inference indicates that the CF/POE under low load mainly plays a role in both internal and external toughening, with POE playing the main role and CF playing an auxiliary role.

**Figure 7:**
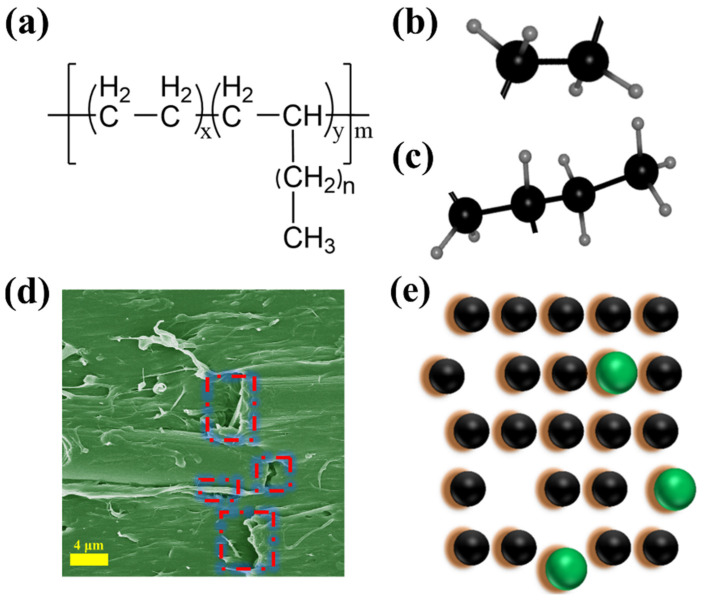
(**a**) The molecular chain structure of POE, (**b**,**c**) the ethylene segment and α-Olefin segment, (**d**) the microstructure of C5 at 2500×, and (**e**) the change model of the microstructure of the composite material after adding CF and being impacted (green sphere represents CF). The red dashed line shows the detachment of CF from the matrix after impact.

Figure 7 shows the structure and composition of POE. The physical crosslinking points in POE are provided by the ethylene segment, which has typical plastic properties and ensures a certain strength. α-Olefins weaken their crystalline regions and enhance their intrinsic elastic properties. The unique molecular structure of POE determines the structure of composite materials. The POE structure has no unsaturated double bonds and obvious short-branched chain characteristics. After melting, it has good processing performance. During the melting process, the composite material is mainly subjected to shear and tensile stresses in the twin-screw transmission. Therefore, the processing temperature in this work is set in the range of 180–185 °C, which is 20 °C higher than the T_m_ of R-PP (around 165 °C). The purpose of the step is to improve the flowability of R-PP and make it stretch the molecular chains as much as possible. The POE with priority unwinding and the PP with partial unwinding will crosslink and fuse recursively under shear force, ultimately forming a microscopic morphology, as shown in Figure 7d. And Figure 7d shows the actual matrix damage caused by CF; Figure 7e shows the voids and possible 3D models.

The significance of Figure 7 is to illustrate the specific impact of the performance improvement of composite materials, which may be explained more intuitively using Griffith’s theory. When a component is subjected to external forces, cracks randomly generate internally, and multiple surfaces are generated between the cracks in different directions. The surface energy of the cracks prevents further propagation of the cracks. When the trend of crack growth coincides with the trend of crack expansion on the surface of the crack, the component enters a critical state, and further expansion of external forces would cause the surface energy to be insufficient to block, leading to fracture. Due to the diversity of composite materials, a reasonable explanation is to incorporate the typical Orowan’s metal fracture theory into the field of composite materials. In addition to stress energy, components subjected to impact can also cause significant deformation that cannot be restored. After being impacted by external forces, CF is pulled out from the R-PP/POE blends, which consumes a certain amount of stress energy and improves the material’s toughness. On the other hand, the key to synergistic toughening lies in the proportion of intrinsic toughening agents, similar to the percolation effect, which only occurs when the proportion of elastomers increases to a certain extent. At the same time, attention should be paid to the tip effect during the diffusion process of force. This is because, during the processing, the CF content is low, and the anchoring effect of the fibers is limited. The arrangement in the matrix may be dominated by anisotropic inclusions and isotropy, making it easier for fractures to develop at weak points after impact. At this time, the elastic body has no time to deform, and the fracture at the connection cannot transmit force to the flexible part, which consumes stress energy. It should be noted that Figure 7 is only a model assumption based on SEM structure and Griffith microcrack fracture theory. Due to the encapsulation and good flowability of POE in SEM, R-PP is entangled in POE and forms a unique molecular physical crosslinking mechanism. A small amount of added CF is evenly dispersed within the system.

**Figure 8:**
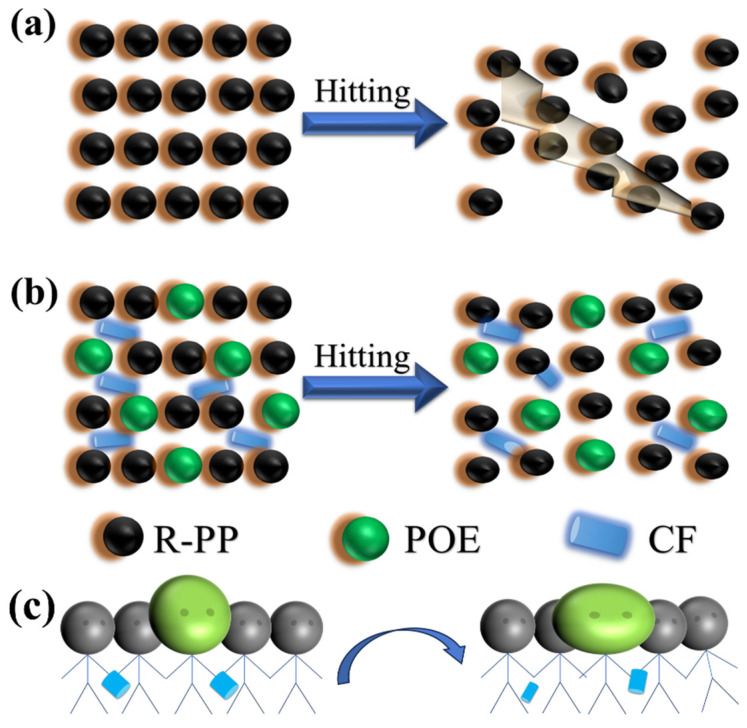
(**a**) Possible site model of R-PP after impact, (**b**) possible site model of composite material after impact, (**c**) possible transformation from the original position to the new position after hit.

To better understand the toughening mechanism of the composite system, a new model was designed, as shown in Figure 8. The reason for the poor impact performance of R-PP is that it is a large-sized spherical crystal molecule with large gaps between spherical crystal molecules and numerous internal defects. When subjected to impact, it was fractured easily at its weak points. The joint toughening mechanism is shown in Figure 8b. In this experiment, we used POE as an external toughening agent and CF as an internal toughening agent (mainly playing a combined role). Accorded to Griffith’s microcrack fracture theory, a reasonable explanation for its impact behavior is that when the composite material is subjected to impact, the elastic propagation behavior of POE consumes a large amount of stress energy, while the fracture and detachment of CF consume a part of the stress energy. Therefore, cracks cannot diffuse from one end of the composite material to the other end, and the material will not fracture. However, combined with this experiment, the mechanical results show that the composite material does indeed have a brittle ductile transition. If the mass fraction of POE is too low, the addition of CF will cause the material to become brittle, increase its hardness and crystallinity, and further fix the position of the molecular chains, making it difficult to move. In addition, excessive addition of CF may not guarantee isotropy, which makes it easier to break under stress and reduces the consumed stress energy. The key to designing such composite materials is to increase the volume ratio of the elastomer to ensure the brittle–ductile transition.

**Figure 9:**
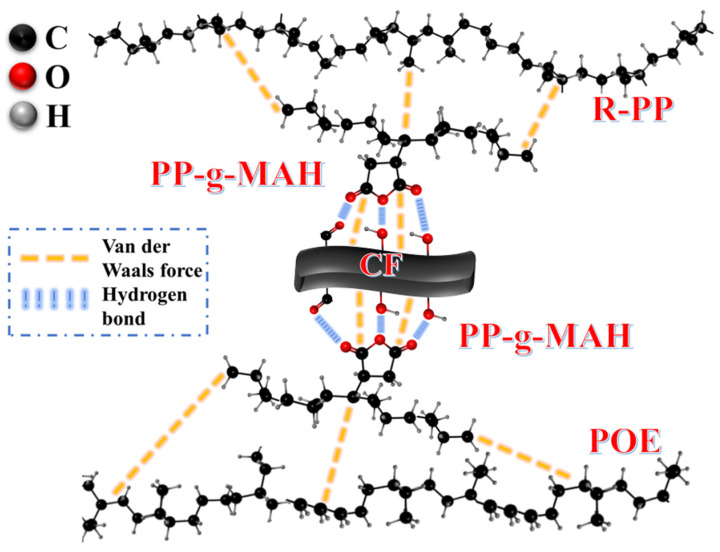
The compatibility mechanisms in R-PP/POE/CF composite material.

In composite material systems, it is always necessary to explain the flow behavior and dispersion, which determine the final performance of the entire process. For this very case, it is necessary to find out why CF can be spirited in composites. Figure 9 shows the possible compatibility mechanisms in composite material systems. Polymer compatibilizers are a type of auxiliary agent that combines two or more incompatible or poorly compatible polymers through intermolecular bonding forces, resulting in the final stable blend. Compared to other additives, maleic anhydride monomer has the strongest polarity and the best compatibility effect. Therefore, in this experiment, PP-g-MAH was chosen as the main additive. Overall, PP-g-MAH introduced strong polar reactive groups into the composite material, resulting in an essential improvement in polarity and reactivity. In composite materials, there is a significant difference in polarity between CF and polymer segments, as well as a significant difference in shrinkage. Therefore, to better leverage the advantages of the material, it is necessary to consider better bonding between multiphase interfaces. PP-g-MAH played the following role as a 1 compatibilizer: on the one hand, the maleic anhydride on its chain segment underwent a chemical reaction with the CF surface, which may be explained by the small amount of oxygen-containing functional groups such as carbonyl (-C=O) or hydroxyl (-OH) remaining on the untreated CF surface [36]. On the other hand, the PP part of PP-g-MAH fused with the substrate R-PP, and it is interesting that the short-branched chain structure of POE was α-Olefins, while PP was a typical α-Olefins; therefore, PP-g-MAH is more likely to fuse with POE simultaneously, and CF is fully integrated with R-PP/POE. The related work can be found in Table 2.

After comparing similar works, it could be found that the enhancement of CF on composite materials is mainly reflected in the tensile strength and bending strength, while the enhancement of POE on composite materials is reflected in the impact strength. After adding POE, the tensile strength of the base PP will decrease [42]. Therefore, using CF/POE simultaneously can obtain high-strength composite materials with both tensile strength and impact strength. In addition, similar work has also pointed out that achieving the brittle–ductile transition point is a key point in designing and processing such composite materials.

## 4. Conclusions

This study constructed an R-PP/POE composite material with CF as the reinforcing phase and improved its interfacial properties and compatibility through PP-g-MAH. The good flowability of POE during processing and the unfolding of chains could wrap the substrate. CF took a role of anchor point for directional insertion into the composite materials, and it could effectively improve the bending and notch impact strength as well as enhance the thermal stability of POE/R-PP. The compatibilizer (PP-g-MAH) dispersed more evenly within the R-PP/POE substrate through hydrogen bonding with a small amount of hydroxyl or carbonyl groups on the CF surface. The addition of CF enhanced its compatibility with the R-PP/POE composites and triggered a joint toughening mechanism.

The addition of 9 wt.% CF successfully improved the heat resistance of the composite material, and the residual carbon content increased by 97.84% after sintering. The composite toughening of POE and CF effectively improved the impact strength of the composite material, with a maximum increase of over 1000%. A reasonable explanation for the impact behavior of the composite was provided by Griffith’s microcrack fracture theory. The elastic propagation behavior of POE could consume a large amount of stress energy, while the fracture and detachment of CF could consume a part of the stress energy, leading to an improvement in impact strength. Furthermore, based on the microscopic morphology after impact, a possible change model was designed to explain the appearance after impact, and it was suggested that the main toughening came from the segmented consumption of corresponding stress energy. The study not only fully designed the possibility of secondary modification based on R-PP but also attempted to combine the synergistic mechanism of internal and external toughening, which is helpful for the plastic industry and its applications. Due to the use of R-PP as the base material, this composite material has certain heat resistance and impact resistance after modification. Therefore, combined with practical production needs, this material can be applied in the manufacturing of disposable medical pallets and PPR water pipes, as well as in building materials and automotive decoration materials that require impact resistance.

## Figures and Tables

**Table 1:** Composition formula of polymer blends.

Sample Code	R-PP/POE/PP-g-MAH/CF [wt.%]
R-PP (A0)	100/0/0/0
R-PP/POE/CF (C1)	50/45/5/0
R-PP/POE/CF (C2)	50/42/5/3
R-PP/POE/CF (C3)	50/40/5/5
R-PP/POE/CF (C4)	50/38/5/7
R-PP/POE/CF (C5)	50/36/5/9

**Table 2:** Comparison Table of related Composite Materials.

Sample [wt.%]	Tensile Strength [MPa]	Impact Strength [KJ/m^2^]	Bending Strength [MPa]	Reference
PP/CF + JF	92.34	2.65 ± 0.5 J/m	44.77	[37]
PP 50/BF 50	7.1	8.9 ± 1.0 J/m	43.3 ± 2.6	[38]
PP/22 SCF	30	285 J/m	-	[39]
PP/30 rCF	-	37	-	[40]
PP/30 RCF	50.62 ± 2.00	12.69 ± 1.14	91.34 ± 1.82	[41]
PP/nEG 20/POE 5	30.0 ± 0.3	10.4 ± 0.3	-	[42]
PP/nEG 20/POE 20	19.7 ± 0.2	22.9 ± 0.3	-	[42]
PP 7/POE-MA 15/TPAS 15	22.6	68.1	-	[43]
R-PP/36 POE/9 CF	29.95 ± 0.57	46.96 ± 1.97	20.01	This work

## Data Availability

Data are contained within the article.

## Supplementary Materials

The following supporting information can be downloaded at https://www.mdpi.com/article/10.3390/polym16070972/s1, Table S1: Results of mechanical testing. Table S2: TGA data of A0 and C system. Table S3: DSC first heating data of A0 and C system. Table S4: DSC second heating data of A0 and C5.
