# Supplementary material for: Carbon Fiber Reinforced Recycled Polypropylene/Polyolefin Elastomer Composites with High Mechanical Properties
Source: Polymers (Basel). 2024 Apr 3;16(7):972. doi: 10.3390/polym16070972 (PMC11013364; doi:10.3390/polym16070972)
Supplement: Supplementary file 1 [file polymers-16-00972-s001.zip › polymers-2912543-supplementary.pdf]

Supplementary Materials

Carbon Fiber Reinforced Recycled Polypropylene/Polyolefin Elastomer Composites with High Mechanical Properties

Jin Wei [1], Abdukeyum Abdurexit [2], Ruxangul Jamal [2], Tursun Abdiryim [1,*], Jiangan You [1], Zhiwei Li [2], Jin Shang [1] and Qian Cheng [1]

Table S1. Results of mechanical testing.

Sample Code	Ultimate Tensile Strength, σM [MPa]	Elongation at Break, εB [%]	Bending Strength [MPa]	Impact Strength [KJ/m²]
A0	32.07±3.18	880.21	38.11	4.10±0.32
C1	29.27±1.49	941.20	13.00	33.39±2.24
C2	30.01±0.80	881.16	16.60	40.60±0.90
C3	30.34±0.77	856.63	18.80	43.27±0.86
C4	31.02±0.78	855.67	19.20	44.14±1.41
C5	29.95±0.57	844.37	20.01	46.96±1.97

Carbon Fiber Technical indicators:

Model: T700-12k,Tensile strength: 4900 GPA Tensile modulus: 230 GPa, Density: 1.75 g/cm^3, Conductivity coefficient: within the third power often, Fiber diameter: 7 um, Section shape: circular, Mesh size: 30-2500,Length to diameter ratio 2:1-70:1,The stretching rate is around 1.7-1.9.

Table S2. TGA data of A0 and C system.

Sample Code	$T_{5\%}$ [◦C]	$T_{50\%}$ [◦C]	Start Point [◦C]	DTG [%/min]	Residue 800 ◦C [wt.%]
A0	433.4	464.4	450	-33.44	5.09
C1	438.2	468.2	452.9	-35.46	1.20
C2	437.6	466.6	451.7	-34.98	3.92
C3	427.3	456.3	441.2	-35.19	3.66
C4	438.3	467.3	458.2	-31.90	10.07
C5	438.0	468.0	452.3	-32.21	8.29

Table S3. DSC first heating data of A0 and C system.

Sample Code	T_{cr1} [◦C]	T_{cr2} [◦C]	ΔH_{m2} [J/g]	T_{m2} [◦C]	X_{C2} [%]
**A0**	**111.81**	**118.13**	**-79.30**	**164.44**	**26.16**
**C1**	**102.33**	**117.93**	**-34.51**	**162.79**	**33.34**
**C2**	**101.35**	**117.98**	**-38.65**	**162.20**	**37.34**
**C3**	**101.17**	**118.10**	**-40.25**	**162.33**	**38.89**
**C4**	**102.33**	**117.93**	**-40.00**	**162.48**	**38.65**
**C5**	**102.11**	**119.33**	**-30.78**	**163.37**	**29.74**

Table S4. DSC second heating data of A0 and C5.

Sample Code	T_{cr1} [◦C]	T_{cr2} [◦C]	ΔH_{m2} [J/g]	T_{m2} [◦C]	X_{C2} [%]
**A0**	**111.97**	**116.99**	**-54.81**	**159.09**	**26.48**
**C5**	**102.84**	**118.89**	**-31.60**	**158.95**	**30.53**
